# Albert Bruschke (1935–2022), a pioneer of coronary arteriography

**DOI:** 10.1007/s12471-022-01756-x

**Published:** 2023-01-24

**Authors:** Norbert van Hemel, J. Wouter Jukema

**Affiliations:** 1 Bunnik, The Netherlands; 2 Department of Cardiology, Leiden University Medical Centre, Leiden, The Netherlands


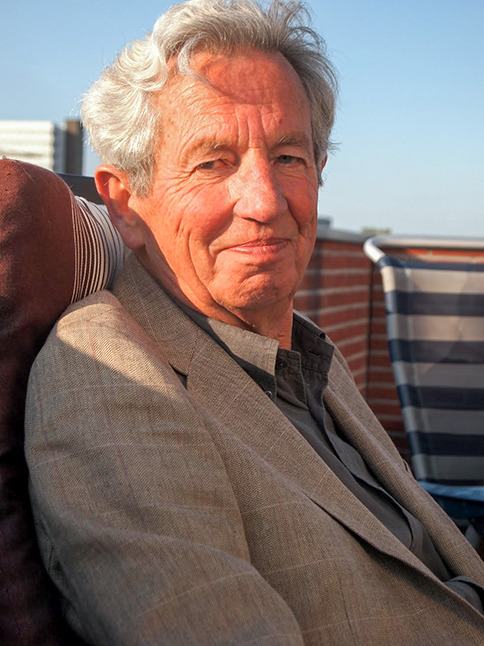


The death of Professor Albert Bruschke on 1 December 2022 was the end of a long, well-designed and prolific medical and scientific life. After his medical training at the Leiden University in Leiden, the Netherlands, followed by an internal medicine internship at the St. Elisabeth Hospital in Tilburg, Bruschke started his clinical cardiology training at the St. Antonius Hospital in Utrecht, where he was supervised by Dr C. L. C. van Nieuwenhuizen. In 1970, Bruschke defended his academic thesis titled “The diagnostic significance of the coronary arteriography” at the Groningen University, at a time when there were no direct surgical or catheter coronary interventions and the mortality rate after acute myocardial infarction was high. His contributions to the understanding of the natural history of coronary artery disease using coronary arteriography were therefore highly relevant.

To explore this topic, Bruschke was appointed special fellow at the Cleveland Clinic in Cleveland, Ohio, USA (1971–1972). In cooperation with cardiologist W. L. Proudfit and roentgenologist M. Sones, he collected a large follow-up angiographic and clinical dataset to construct survival curves. This resulted in his famous publications on the progression of coronary artery disease in *Circulation* in 1973. In 1982, these publications were classified as “Citation Classics”, after which their content was often applied in the management of patients with coronary disease.

From 1973 to 1985, Bruschke chaired the Cardiology Department of the St. Antonius Hospital and spread his broad insights into coronary arteriography through many clinical studies on the diagnosis and surgical and later transcutaneous catheter revascularisation of patients with coronary artery disease. To avoid the image of being a clinical track–only department, he underscored the development of new cardiology fields such as cardiac nuclear studies, echocardiography and cardiac arrhythmia. This also included close cooperation with the former Medical Physics Institute in Utrecht, which resulted in many clinical and epidemiological electrocardiography and vectorcardiography studies and academic dissertations.

After Bruschke was appointed professor and head of the Cardiology Department at the Leiden University Medical Centre (LUMC) in 1985, his main activities were teaching as well as initiating and supervising scientific studies. Until his retirement in 2000, he supervised 22 academic dissertations, reviewed manuscripts for several journals and wrote books about arteriography and coronary artery diseases. He contributed strongly to the Dutch Regression Growth Evaluation Statin Study (REGRESS) of the Interuniversity Cardiology Institute of the Netherlands (currently, the Netherlands Heart Institute), showing the significance of quantitative arteriography to assess the clinical value of lipid-lowering medication on the natural course of coronary artery disease (published in 1979).
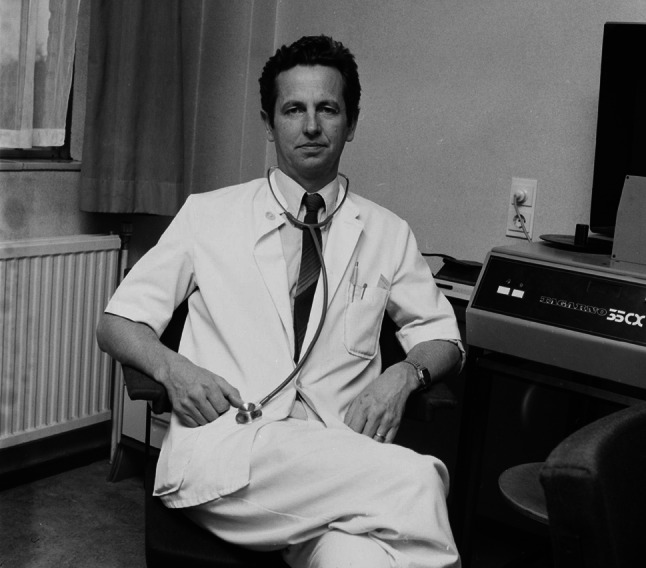


Because of his expertise, Prof. Bruschke, not surprisingly, became a Fellow of the Society of Cardiac Angiography, a Fellow of the College of Chest Physicians and a Fellow of the American College of Cardiology. He was also a founding father of the European Society of Cardiology. In the Netherlands, his reputation in coronary arteriography led to chairing many committees in this field (1973–1978). He also represented the cardiology community in several medical organisations (1980–1982) and chaired the Netherlands Society of Cardiology (1983–1985).

After his retirement, Bruschke continued several activities at the LUMC, as a teaching professor for the junior doctors and as a supervisor of research papers, amongst others. His ongoing intensive correspondence with and commitment to his favourite Cleveland Clinic for more than 40 years were recognised with the Service Award of the Cleveland Clinic Foundation Alumni Association in 1999.

Prof. Bruschke can be characterised as someone with high standards of discipline and fairness; his scientific approach and thorough verification of data collection were well recognised. However, his attitude could readily cause conflict with individuals who had an easy or superficial professional approach. This attitude became apparent not only in the clinic, but also in his free time. Bruschke was a fanatic Atlantic race sailor: once, his team won the Channel Race.

Staff members of the Cardiology Department at the St. Antonius Hospital in Nieuwegein/Utrecht in 1970–1985 and those of the Cardiology Department at the LUMC in later years gratefully acknowledge Prof. Bruschke’s inspiration and expertise. This also holds true for the numerous cardiologists trained in the abovementioned institutions who experienced his impressive clinical knowledge and drive. The patient with coronary artery disease can also thank Dr Bruschke, because his papers have strongly contributed to improved management of this disease, which was further explored and updated by other cardiologists. In 2004, Prof. Bruschke was appointed honorary member of the Netherlands Society of Cardiology. One of the reasons for receiving this award was his important contribution to the care of the coronary patient.

The history of the fruitful, dedicated and well-organised life of Albert Bruschke with his characteristic behaviour and comments will remain in our heart and memory. We wish his wife Gon and family the strength to cope with his passing away.

